# Employability skills for MICE management in the context of ICTs

**DOI:** 10.1371/journal.pone.0271430

**Published:** 2022-07-13

**Authors:** Xiao Liu, Randy Seevers, Hongyi Lin

**Affiliations:** 1 School of Cultural Industries and Tourism, Xiamen University of Technology, Xiamen, China; 2 Research Centre for Cultural Industries, Social Science Research Base of Fujian Province, Xiamen, China; 3 College of Education, University of Houston-Clear Lake, Houston, TX, United States of America; 4 School of Optoelectronic and Communication Engineering, Xiamen University of Technology, Xiamen, China; Universidad Rey Juan Carlos, SPAIN

## Abstract

Information and Communication Technologies (ICTs) applications have become a vital part for MICE industry. MICE higher education is expected to provide their graduates with essential management knowledge and ICTs operational skills to meet the industry demands on the rise. This empirical study investigates the perceptions of employability skills for MICE management in the context of ICTs. Based on the questionnaire (n = 95), an initial 16 employability skills are proposed and the underlying dimensions are explored. The skills of communication, innovation, organizing and coordinating, market promotion, planning, project implementing, crisis management, proficiency in English and operation management are perceived as of great importance. Four categories of employability skills are analysed: Core Generic skills (CGS), Communicative Expression Skills (CES), Practical Hands-on Skills (PHS) and MICE Professional Skills (MPS). This study is crucial as it helps to identify the level of importance and dimension of employability skills for MICE management. For both academia and industry, the results of this study are useful to provide critical skills for multi-skilled and competitive employees for their future success.

## Introduction

The diffusion and development of Information and Communication Technologies (ICTs) across various economic areas have brought up new demands for employee’s competences and skills [1]. The emergence and use of ICTs and digital technologies are transforming the world and the government’s role and service, facilitating the development of sectors such as education, agriculture, and service [2]. The evolution of ICTs and its integration in the manufacturing sector is altering traditional industries, stimulating the development of new business models, services and behaviours in tourism industry [3]. MICE industry is a subtype of tourism and hospitality industry, which belongs to the service industry [4]. It is commonly believed that there has been a significant increasing of ICTs application in the MICE industry. ICTs and MICE industry have made a synergistic effect, which increases service efficiency and improve service experience for MICE stakeholders [5]. It represents a technological and paradigm shift in the MICE industry which arouses an upgraded industry form of “Smart MICE”. Smart MICE is considered as an innovative high-tech service industry which is built on the infrastructure of ICTs to guarantee the sustainability of MICE sectors. Smart MICE industry shows the integration effect for data exploration, collection, analysis, utilization, and aggregation on user experience, service quality, and operational efficiency improvement [6, 7]. ICTs are indispensable to facilitate routine business tasks in conferences, exhibitions and shows [8]. Advanced technologies such as Web 2.0 [9], mobile technologies [10], Virtual Reality (VR) [11–13], Augmented Reality (AR) [14, 15] are frequently used for users’ feedbacks and experience in the conference and exhibition centers and virtual shows. In addition, Radio Frequency Identification (RFID) is widely applied for identifying and tracking users’ location in the exhibitions [8, 16]. According to the report entitled “Best Practice in Digital Innovation”, MICE industry has experienced web applications, web-based applications, customer relationship management projects, social media, mobile applications, overall solutions for data management, IT departments or companies, service cloud platforms, and artificial intelligence and AR, when it comes to digital era [17]. Another report focusing on the development and outlook of the global exhibition industry entitled “UFI Global Exhibition Barometer” shows that the topics of “Impact of Digitization”, “New Digital Products” and “Competition with Other Media” are still among “most important business issues” in MICE industry [18]. All of aforementioned changes in MICE industry focus on improving existing online services, adding value to the users’ experience, and providing solutions for conference organizers and venue managers.

As is claimed by International Congress and Convention Association (ICCA), MICE industry is one kind of tourism and hospitality in which a variety of groups get together for special purposes. The activities of MICE are covered by event tourism [4, 19]. The relationship between MICE and tourism & hospitality shows that generic competences of graduates in tourism and hospitality can be used as the reference for MICE education. High calibre and multi-skilled graduates with creativity, flexibility and adaptability [20, 21], soft skills such as interpersonal relationships with customers and employees, and oral and written communication [20], teamwork, time management, communication skills, knowledge acquiring and application skills, professional skills, expression skills, and ICTs management and application skills [22] are essential and popular competencies in tourism and hospitality management education. MICE students are expected and desired to develop with project management, working with audiences, promotion and public relations, fundraising and development, written and oral communication, willingness to learn, reliability, commitment, flexibility, self-motivation, and time management [23], event document handling [24] and communication, intercultural and MICE professional competencies [25].

Given the technological revolution, the challenge MICE industry must face concerns how to train skilled professionals to fulfil the needs of MICE companies in the context of ICTs. While ICTs expands the space of the curriculum in MICE, it is not clear which of these employability skills can be crucial and necessary for graduates. Issues on employability skills in MICE education should always be a major topic of concern for researchers in the further development of MICE, tourism and other related industries. This study addresses the lack of relevant research by investigating MICE industry perception of employability skills in the background of ICTs. Its purpose is to highlight and evaluate the level of importance for multiple MICE management employability skills and to explore its underlying factor structures. This study discusses what should be provided and taught, and how these can be cultivated in a better way. The results of prioritization and supplements of skills for future MICE professionals will provide theoretical references and practical guidance to fill the gap between the students’ preparation and industry expectation.

## Literature review

### ICTs applications in MICE sector

As the technological foundation for “Smart MICE”, ICTs’ application in the MICE industry can be summarized into three aspects: infrastructure and framework, feedback and experience, identifying and tracking. In the first aspect, Wi-Fi, 5G and IoT are frequently used to provide a basic technological environment for other technical tools. Wi-Fi and 5G have been an irresistible trend in most of conferences, conventions and exhibition centers, which can promote further communication and interactions among users and products [14], while IoT acts as a platform to generate data and support real-time exhibit shows [26]. In the second aspect, technology adoption with Web 2.0 enables social media and cloud computing to interact [27]. Mobile technologies, which consist of Android, iOS and Windows, can facilitate the implementation of VR and digital exhibitions [10]. VR business applications are widely used in conferences and exhibitions such as desktop sharing, webcasts, 3D environments and multi-day conferences [13] to help people who have no time to be on-site or who are unable to attend [11, 12]. AR-technology smart exhibition system based on digital devices enables visitors to obtain more detailed information [15]. As for the third aspect of technologies to identify and track attendees, booth recommendation systems are designed based on RFID and Quick Response (QR) code to collect data by scanning readers and tags [8, 16]. As a type of RFID technology, Near-Field Communication (NFC) is a contactless communication technology which has been developed into NFC-based O2O service platform to send and receive data, and foster interconnection among partners in the exhibitions [28].

### Generic, professional and ICTs competences for MICE education

It is common for the application of competences as a general standard to evaluate the educational programs and activities. According to European Credit Transfer System (ECTS) User Guide, competence represents “the proven ability to use knowledge, skills and personal, social and/or methodological abilities, in work or study situations and in professional and personal development” [29], which can be generic or subject-specific. Generic competences are usually defined as the sum of knowledge, capacity and character, with the characteristics of transferability and integration from work and social life [30]. Scholars also capitalize on employability instead of competence. Employability is a potential for future success in the graduates’ chosen field, which is determined by understanding, skills, efficacy beliefs and meta-cognition [23]. Students’ employability mainly depends on individual factors, market labor and practices in organizations [31]. The results of 36 competences’ rank order for management trainees showed that soft skills such as interpersonal relationships with customers and employees, and oral and written communication were most essential competences in the views of graduates and hotel managers in Greece [20]. Teamwork, time management, communication skills, knowledge acquiring and application skills, professional skills, expression skills, and ICTs management and application skills were evaluated as the most important skills among 30 generic competences of graduates by tourism employers in Balearic Islands [22]. The need for higher educational institutions including universities and colleges was emphasized to embed ICTs skills into tourism curricula [32].

As tourism and hospitality industry professionals need to evolve in their trainings and skills, MICE and Event management professionals are desired to be trained in employability skills development as well [33]. MICE sector covers a wide range of activity management such as special event management, on- and off-premise catering, and exposition management [34]. Typically, MICE students are expected to transfer multi-facet, comprehensive and adequate competences and experience to their future work environment. Project management, working with audiences, promotion and public relations, fundraising and development are the desirable vocationally-specific knowledge for the applicants in arts and event sector. Moreover, written and oral communication, willingness to learn, reliability, commitment, flexibility, self-motivation, and time management are the expected transferable skills by employers [23]. “Event Document” was evaluated as the most important course among 27 event management courses in one investigation in Shanghai [24]. For international MICE professionals, content-based competences were ranked priorities by experts through an analytic hierarchy process approach in the aspects of communication, intercultural and MICE profession [25]. The aforementioned research studies have examined the perceptions and expectations of tourism, hospitality and MICE employment generic competences and skills for graduates. Most of these studies focused on rank orders for importance of competences and skills [20, 22, 25], and importance of the courses in curricula system [19, 24, 34] reported by the industry employers and experts. Some study provided data from the perspective of graduates [23].

Although there is no generally agreed-upon definition of ICTs competency, educators and industry employers commonly recognize and highlight the importance of ICTs for various disciplines. University students should acquire IT proficiency in software application such as Microsoft Office, email, database management and statistical analysis [35]. In one study, 20 generic skills required for ICT professionals are assertiveness, teamwork, company loyalty, commitment to learning, self-control, creativity, empathy, flexibility, taking the initiative, innovation, leadership, empowerment, task-oriented, customer-oriented, analytical thought, problem solving, communication, negotiation, planning and information research [30]. Cheung and Law [36] reported the 6 crucial dimensions of IT competence for Hotel and Tourism Management (HTM) graduates, including information preparing, information handling, communications, applications of software, management information systems, and evaluating. In arts and event management, word processing and spreadsheets are two most essential vocationally-specific knowledge or skills, while databases, presentation and web design are most desirable among all software applications [23]. Therefore, ICTs-related courses such as management and administrative software tools [36], and digital design and management information system [19] are recommended to be included in the HTM and MICE curricula of operation-focused training programs.

### Skills gap between industry and academia in the context of ICTs

Skills gaps and mismatches between industrial application and higher education have become prominent in many academic programs, including hospitality and tourism [21, 36], MICE [37] and ICT [30]. Negative perception of the policy in higher education for one country is caused by the issue of unemployed graduates and the obstacle of acquiring workforce [31]. The knowledge of ICTs continues to evolve and update in the information age. As a result, university graduates face a dilemma that what they have learned in school shows low relevance with their future work [38]. Nonetheless, the expectation of employers pays more attention to transferable knowledge and practical skills to solve the actual problems in the workplace [36]. The obstacles to students to meet the needs after graduation are the gap between the job skills and the skills required in the workplace [39]. It is found that effective communication and collaboration between academia and industry is inadequate and insufficient [36]. Employability skills become important for they can facilitate graduates to change their jobs among organizations, which remains one significant missing linkage between university education and industry work [39]. With the advancement in technologies, especially in ICTs, access to e-learning course contents has contributed to the employability skills for graduates around the world. In India, Higher Education Institutions are facing a transition phase in the rapidly changing technological environment, in which a diversified and novel approach towards professional skills training is needed [40]. Researches show that the major challenge in tourism is the gap between the supply and the demand both in Turkey [41] and China [42]. Current study showed that more than 40% students hesitated to stay in tourism or worked in other industry after graduation in Northern Cyprus [43].

The goal of MICE education is to provide well-educated and eligible industry specialists for the MICE industry [24]. Continuous MICE technological transformations provide a driving force for cultivating specialized and skilled professionals, and keeping the pace with emerging industry trends. Academic in MICE education is facing two challenges: (a) how to educate students in this dynamic and varied workplace environment, and (b) how to make the connection with practitioners in the industry [37]. MICE industry professionals and stakeholders expect the graduates in the four-year baccalaureate program to acquire not only with traditional management competencies such as marketing, organizing, coordinating, interpersonal relationship and crisis management, but also skills related to ICTs. However, training for MICE in higher education seems lagging behind the developments of MICE industry upgrade. Educators in tertiary education have not been able to update their teaching curricula and materials to accommodate the technological enhancement in MICE industry in time. Compared to MICE graduates, students with educational background of computer science or related area will be popular in MICE industry, as they are much easier to acquire and utilize technological knowledge and skills into MICE sector. In other words, the problems facing “Smart MICE” today is the skills gap between what the MICE industry expects and what higher education delivers. It is urgent for educators in MICE to adjust the goals in the aspect of updated and targeted knowledge to supply the workforce with skilled professionals for the industry.

## Research methodology

### Questionnaire development

This study used a questionnaire as the data collection tool. The questionnaire consisted of questions on MICE employability skills and demographic questions in Chinese. The items relate to the MICE employability skills were adopted from the questionnaires used in previous studies [20, 21, 23, 30] and drawn from the teaching experience of first author who has taught in the MICE undergraduate program in one university in China since 2011. The items on employability skills assessed the level of importance of each skill for MICE graduates in the background of ICTs on a five-point Likert-type scale with the value of 1 as “very unimportant” to 5 as “very important”. To ensure the content validity of the questionnaire, a group of MICE industry experts and academic professors (n = 3) were asked to evaluate each item for feasibility, logic and reading fluency. A pilot study was then conducted by asking 10 students who would not participate in the actual study to fill out the questionnaire. The students’ feedback was used to further improve the content validity of the questionnaire. The number of items in the final version was still 16 with minor adjustments for clear language expression.

### Data collection

The research was conducted over two months in China. A convenience sample of 120 was selected from the students majoring in MICE Economy and Management and employees, who had been engaged in the event, conference, and exhibition industry for our investigation. In order to achieve sufficient minimum sample size for our research, the questionnaires were distributed to ensure a high response rate, and in case of inconsistent and incomplete feedback by both online and on-site. Having sufficient samples and a high response are recommended, however, the response rate at least 60% is acceptable. All the on-site surveys were collected in school classrooms in paper edition. Of the 120 distributed questionnaires, 95 were returned (on the spot or online), complete and usable for further analysis, with the valid response rate of 79.2%. Obvious regular answers were deemed invalid. For instance, all the answers were 1 or 5. Online questionnaires responded less than 30 seconds were also considered as invalid, because it is too short for participants to make a reply with careful consideration.

### Results and analysis

The researchers deployed SPSS 26.0 for data analysis. Descriptive analysis was used to show social-demographic characteristics of the survey sample and their perceptions of the importance for MICE employability skills. Exploratory Factory Analysis (EFA) was used to measure the construct dimensions of MICE employability skills. Independent sample t-test and ANOVA were conducted to obtain a better understanding for the significance of the identified factors on different demographic variables.

Demographic data showed that 81.1% of the respondents were female. The majority of the respondents were in the age range of 18–24 years old group (96.8%). Most of them have the educational background of MICE-related major (82.1%), while 17.9% respondents are with the education background of other disciplines. In terms of work experience in MICE, most of the respondents were less than one year (91.6%). Table 1 presents demographic characteristics for the full respondents.

**Table 1:** Social-demographic information of the survey sample (N = 95).

Demographic Characteristics	Description	Frequency	Percent (%)
Gender	Male	18	18.9
	Female	77	81.1
Age	<18	0	0
	18–24	92	96.8
	25–30	2	2.1
	>30	1	1.1
Do you have a degree in MICE (4-year)	Yes	78	82.1
	No	17	17.9
Years of work experience in MICE	Less than 1 year	87	91.6
	1–3 years	7	7.4
	3–5 years (3 years excluded)	0	0
	more than 6 years	1	1.1

Table 2 shows the results of descriptive analysis for 16 MICE employability skills. It also reveals the level of importance for these skills. First, the overall mean value of all 16 attributes is 4.07, compared with 5.00 as the highest. Taking 4.00 as the benchmark, the mean values of top 8 attributes are above 4.00. The results imply that respondents perceived most of these skills as of considerably higher importance in general. Second, “interpersonal relationship skills” is considered as the most important among 16 listed skills. This finding is consistent with the research of Beaven and Wright [23] which found written and oral communication were the most transferrable skills that employers expect for the arts and event management graduates. Third, attributes of “interpersonal communication skills”, “innovation skills”, “organizing and coordinating skills”, “market promotion skills”, “planning skills” and “project implementation skills” are ranked in the top 5 of the list in Table 2 (organizing and coordinating skills and market promotion skills are in the same rank). The MICE is composed of four aspects (meetings, incentive travels, conventions and conferences, exhibitions) with multiple parties for group business activities [5]. The industry experts and professionals tend to give priority to business activities [24]. Then, skills on communication, innovation, organization, coordination and planning are perceived with relatively high importance according to this result. Fourth, scores of ICTs-related skills such as “internet thinking skills”, “data analysis and management skills”, “online marketing skills” and “computer operating skills” are above 3.60. This finding implies ICTs-related skills attract inadequate attention up to now.

**Table 2:** Levels of importance for MICE employability skills.

Rank order	Attributes	Mean	Standard deviation	Variance
1	Interpersonal communication skills	4.58	0.594	0.353
2	Innovation skills	4.39	0.719	0.517
3	Organizing and coordinating skills	4.36	0.728	0.53
3	Market promotion skills	4.36	0.771	0.594
5	Planning skills	4.31	0.773	0.597
6	Project implementation skills	4.27	0.691	0.477
7	Crisis management skills	4.13	0.854	0.729
8	Proficiency in English	4.08	0.883	0.78
9	Operation management skills	4.04	0.728	0.53
10	Professional learning skills	3.99	0.917	0.84
11	Data analysis and management skills	3.97	0.831	0.69
12	Internet thinking skills	3.96	0.967	0.934
13	Customer service skills	3.91	0.759	0.576
14	Online marketing skills	3.74	0.936	0.877
15	Computer operating skills	3.64	0.743	0.551
16	Art and Design skills	3.39	0.829	0.687

The Cronbach’s alpha coefficient of the 16 items is 0.859, which is much higher than the usual benchmark of 0.7 [30, 44]. It represents that the scale has a good reliability of internal consistency. The result of Kaiser-Meyer-Olkin (KMO) measure of sampling adequacy is 0.783, while the Bartlett’s test of sphericity is significant (p <0.05). The results assessed the factorability of the data and provide support for the following exploratory factor analysis (EFA) [44]. The perceived importance of 16 attributes was analysed by EFA, using the principal component analysis to determine the underlying dimensions with varimax method for factor rotation [44]. The application criteria in this study for EFA were the following: Eigenvalue > 1, cut-off points > 0.50 and cross-loading > 0.10 [21]. Thus, the items of “Innovation skills”, “Organizing and coordinating skills”, “Crisis management skills” and “Computer operating skills” are deleted. This study identifies four underlying dimensions of MICE employability skills in the background of ICTs in China (see Table 3). These four dimensions are:

- Factor 1: Core Generic Skills (CGS) (Eigenvalue = 4.306, Cronbach α = 0.833)
- Factor 2: Communicative Expression Skills (CES) (Eigenvalue = 1.646, Cronbach α = 0.627)
- Factor 3: Practical Hands-on Skills (PHS) (Eigenvalue = 1.183, Cronbach α = 0.633)
- Factor 4: MICE Professional Skills (MPS) (Eigenvalue = 1.035, Cronbach α = 0.660)

**Table 3:** EFA for MICE employability competences and skills.

	Rotated factor loading			
Items	CGS	CES	PHS	MPS
Customer service skills	**0.607**	-0.147	0.263	0.289
Proficiency in English	**0.546**	-0.300	-0.146	0.375
Art and Design skills	**0.675**	-0.013	0.185	0.248
Internet thinking skills	**0.828**	0.212	0.112	0.112
Data analysis and management skills	**0.758**	0.234	0.183	-0.078
Online marketing skills	**0.815**	0.172	0.099	0.094
Interpersonal communication skills	0.151	**0.839**	0.071	-0.047
Market promotion skills	0.064	**0.790**	0.122	0.167
Operation management skills	0.193	0.067	**0.821**	0.050
Project implementation skills	0.139	0.159	**0.801**	0.131
Professional learning skills	0.379	-0.099	0.318	**0.705**
Planning skills	0.076	0.230	0.035	**0.868**
Cronbach’s alpha	**0.833**	**0.627**	**0.633**	**0.66**
Eigenvalue	**4.306**	**1.646**	**1.183**	**1.035**
Variance (%)	**27.313**	**13.839**	**13.489**	**13.436**
Cumulative variance (%)				**68.078**

Factor 1, CGS, consists of 6 items. The item with the highest factor loading is “internet thinking skills” (0.828) and followed by “online marketing skills” (0.815). The Cronbach’s α for CGS is 0.833, higher than the cut-off point of 0.7. It indicates a scale of high reliability. Firstly, one of 6 items deals with basic management skill, which is “customer relationship skills”. This skill is of great significance in line with the previous findings. For example, it showed that “managing guest problems with understanding and sensitivity” and “developing positive customer relations” were ranked in the top 2 among 36 competences for hospitality management as reported by hotel managers [20]. The item of “event customer service and management” was ranked 4th among 27 event management courses by industry professionals [24] and “customer oriented” was reported as a generic skill by ICT professionals [30]. The relationship with customers allows employees to analyse users’ needs and customize the services which will be offered in tourism [3]. Therefore, “Customer service skills” is one of CGS in MICE industry. Secondly, the item of “proficiency in English” belongs to CGS for the reason that this survey was conducted, an ESL country. Business English is a required subject for many university students in MICE. It is no surprise that respondents in this study consider the proficiency in second language as core generic competences and skills. This result is consistent with Dhiman [21] who discovered that the item of “proficiency in English” belonged to the factor of “basic understanding and skills” for tourism graduates in India. Zeng and Yang [24] noted the item of “English for Convention and Exhibition” was ranked in the top 5 of the list for the importance of 27 event management courses. Thirdly, one of the services provided for visitors in MICE industry is booth design and construction. Therefore, the item of “art and design skills” is an essential technological ability for CGS. Moreover, it is interesting to note that CGS includes three items which are related to ICTs skills. These three items are “internet thinking skills”, “data analysis and management skills” and “online marketing skills”. This finding is consistent with the research done by Dhiman [21] and Bilgihan et al., [45]. “ICT and computer ability”, “ability to use information”, “ability to handle information” and “technical and quantitative ability” were the attributes of “basic understanding and skills” for tourism graduates in India [21]. “Understanding the latest IT trends in the industry”, “analyse numerical data with computers” and “online marketing tools” were the IT skills of great importance reported by the respondents [45]. The technological skills on the collection of data and their analysis were identified and required in tourism education [3].

Factor 2, CES, contains two items, which are “interpersonal communication skills” and “market promotion skills” with 0.627 Cronbach’s α coefficient. The item with the highest factor loading is “interpersonal communication skills” (0.839). Generally, it is recommended that no fewer than three items for every factor. However, a scale in measuring more than one factor may require as little as two items per factor [46]. The result of only two items per scale also existed in Gosling et al., [47] for the Ten-Item Personality Inventory of Big-Five personality scales. As for the appropriate cut-off points of the reliability, there is much debate among researchers. If Cronbach’s α is between 0.5 and 0.7, it shows moderate reliability for the sub-scale [48]. In an exploratory research, Cronbach’s α may decrease to 0.6 [49]. Thus, Cronbach’s α over 0.6 is considered acceptable [50]. In a study conducted by Christou [20], competencies of “communicates effectively both written and orally” were ranked the fourth importance, with the mean value of 4.66, by hospitality management trainees. Dhiman [21] also noted that “written communication skills” and “oral presentation skills” were the basic skills in tourism management employability. Zeng and Yang [24] found that “event marketing” was ranked in the top five of selected courses, suggesting that students need this course to develop their specified promotion skills.

Factor 3, PHS, comprises two items of “operation management skills” and “project implementation skills”, with 0.633 Cronbach’s α coefficient. The item with the highest factor loading is “operation management skills” (0.821). This factor identifies the trainees’ capabilities in conference and exhibition operation. According to Zeng and Yang [24], “project management” was ranked in the top five of great importance of selected courses in event management curriculum. Christou [20] also found that the competence of “identifies operational problems” was the 6th in the rank order of 36 competencies for hospitality management by hotel managers. Moreover, CES and PHS both reflect the employability skills related to entrepreneurship and business administration. Entrepreneurship has been introduced as one key course in the curriculum of multiple college and universities across the world [40].

Factor 4, MPS, includes 2 items of “professional learning skills” and “planning skills”, with Cronbach’s α coefficient of 0.660. These items all share one common characteristic in that they all have direct relations with MPS. The item with the highest factor loading is “planning skills” (0.868). Obviously, “professional learning skills” and “planning skills” are two critical competences that are required for personnel and staff in MICE industry. This result corroborates with the findings in Moscardo and Norris [37] and Zeng and Yang [24]. Planning was highlighted as one of the implications in the area of conference and event management, as staff needs to take more time during the initial planning stage [37]. Moreover, “event planning” was ranked 2nd among 27 event management courses by industry professionals [24].

The results of Independent sample t-test and ANOVA show that: (1) no significant difference is found for four factors in terms of gender (see Table 4). (2) Statistically significant difference is found for Factor 2 (CES) by MICE degree background (see Table 5). (3) ANOVA is failed to carry out for the reason that some cases are fewer than two. This happens on the demographic variables such as age and working experiences. Another interesting finding is that there is no significant difference for Factor 1 (CGS) in terms of MICE degree background, but there is significant difference for Factor 2 (CES). This result implies that whether the respondents have a MICE degree or not, it will not affect the abilities training in the aspect of core generic skills. It also shows that ICTs-related skills, which are included in CGS, are perceived of great importance as generic competence by current undergraduates and graduates from in MICE and in other majors. But for CES, the perception is significantly different between respondents with and without MICE degree. This can be explained that postsecondary education provided a variety of courses such as event customer service and management, consumer psychology, mass-communication [24], etiquette and communication skills [19] for MICE major students.

**Table 4:** T-test for the influence of gender on the skills of MICE students.

Dependent variables	Levene’s test for equality of variance		T-test for equality of means	
	F	Sig.	t	Sig.
CGS	0.123	0.726	-1.847	0.068
CES	2.949	0.089	1.014	0.313
PHS	0.009	0.926	0.866	0.389
MPS	3.648	0.059	-0.945	0.347

Note: *p<0.1; **p<0.05; ***p<0.01

**Table 5:** T-test for the influence of MICE degree background on the skills of MICE students.

Dependent variables	Levene’s test for equality of variance		T-test for equality of means	
	F	Sig.	t	Sig.
CGS	0.454	0.502	-0.539	0.591
CES	7.051	0.009***	-3.941	0.000***
PHS	0.116	0.734	0.605	0.547
MPS	0.389	0.534	1.957	0.053

Note: *p<0.1; **p<0.05; ***p<0.01

## Conclusions

This study examines the importance of employability skills in the background of ICTs and investigates any significant differences among respondents’ demographic variables and underlying dimensions of employability skills for MICE industry.

Of 16 skills in MICE employability, 9 skills are scored higher than 4.00. The top 5 skills are “interpersonal communication skills”, “innovation skills”, “organizing and coordinating skills”, “market promotion skills” and “planning skills”. The findings of this study corroborate with the results of previous studies on employability in MICE [23, 24] and in tourism [20, 21, 51]. Numerous ICTs applications have been employed in various aspects of MICE operations. In addition to regular ICTs skills such as using word processing, spreadsheet and email systems for computer operations, respondents considered some specific skills important, including analysing data with computers, keeping up current latest ICT trends, and using online marketing skills. These skills were also reported in Bilgihan et al. [45] who hold that graduates of hospitality school were perceived to have ICTs-related knowledge.

The results also indicate that CGS, CES, PHS, and MPS are four dimensions of employability skills required for success in the career of future MICE business world. In checklist of CGS, two items of them are “internet thinking skills” and “data analysis and management skills”, which are also supported by the views of “ICT and computer ability” [21], “ability to use information technologies” [22], “vocationally-specific knowledge or experience on software applications” [23], “information handling” and “applications of software” [36], and “using computer effectively” [51]. As ICTs have been integrated into MICE sector and affected all the customers, employers, and employees, ICTs-related skills become generic and essential competencies for MICE students. This study reveals that ICTs-related skills such as internet thinking skills, data analysis and management skills belong to Core Generic Skills (CGS).

## Implications, limitations and future research

### Implications for academia and industry

This study conducts an exploratory analysis of MICE employability skills and its factors in the context of ICTs. The findings of this study could serve as a pointer for further studies in MICE education, contributing to developing a deeper understanding of MICE employability skills. The results will provide industry professionals, faculty members, and students with more valuable information about the employability skills for MICE management, and could further guide the application of ICTs in MICE education in the future.

With the increasing ICTs application in MICE industry, the need for multi-skilled, qualified and well-trained employees is imperative. Graduates are suggested to equip themselves with both soft and hard skills for more job opportunities in MICE market. This study is meant to acknowledge the role of ICTs in the development of MICE students’ employability skills and emphasize the importance of keeping pace with current technological trends in MICE education. The results of this research recommend a close industry-education partnership to overcome the gap between academia and industry.

Firstly, employability skills development in MICE education is part of ability training and learning process. It is necessary for every employee in MICE sector to master the competences and skills in CGS, CES, PHS and MPS. In turn, this can help to develop a broader framework of how to provide better preparation and more flexible labor force in the background of ICTs. Similar to ICTs, Digital technologies (DTs) include information, computing, communication, and connectivity technologies. Therefore, digital business capacities are required to build and sustain by hospitality and tourism, as well as MICE organizations. Strategic DTs competencies will attach greater importance to higher-level managers, whereas technical DTs competencies are more suitable for lower-level managers [52]. The skills gap between industry and academia will be narrowed and filled by providing tailor-made courses in the field of MICE industrial campaigns, skill development programmes, and management development programmes for the college graduates [40]. Programs should focus on the training in both scientific observation and academic research for students to bolster their industry perceptions [53]. Only in this way can educators avoid a “one size fits all” approach for the MICE labor markets and propel the MICE industry to a new stage with the injection of innovation and technological know-how.

Secondly, the outcomes of this study can be treated as a starting point for the redesign and reconstruction of higher education course curricula in MICE management. The development of the students’ ICTs-related skills should receive special consideration as the ever-changing nature of ICTs necessitates the continuous update of MICE technological application curriculum [36]. Taking New York University as the example, the course of “Meetings Technology” helps students to understand and apply up-to-date MICE technologies. Exhibition and show technologies, such as virtual reality, laser exhibition technology, holographic interactive projection, and spatial interaction techniques [54] could gradually be introduced into MICE course contents within a reasonable range. Besides direct ICTs-related knowledge, ICTs-related skills such as using spreadsheet, email system, word processing, presentation, and data analysis [45] must be taught to equip the graduates with appropriate technological know-how [36]. Based on ICTs, big data technologies focus on information analysis and hidden facts extraction with data mining, data handling and data prediction [55–57]. Curricula framework will be expanded and integrated with more specific understanding of ICTs and DTs competencies by curricula designers [52]. An efficient curricula system, including skills development related to ICTs and DTs, should be established between academia and industry in order to meet the rising challenges of the rapidly-developing MICE industry.

Thirdly, the teaching modes for MICE major would be changed with advancement in ICTs. Educators should consider integrating ICTs into students’ learning style for they have been immersive in ICTs and satisfy their whole lives in tourism [58] and MICE education [59]. ICTs represent the tools and technologies applied to produce, disseminate, preserve, manage, and communicate the information during the process of teaching and learning. Therefore, the incorporation of ICTs in learning activities could be an effective solution to enhancing the students’ skills [60]. The teaching activities could be carried out with the combination of particular courses. For example, the course of “Customer relationship management”, related to customer service skills, is suggested to develop by lectures, hands-on activities and virtual simulations. The course of “Operation management” and “Marketing”, relevant to operation management skills, online marketing skills and market promotion skills could be conducted by seminars, project-based models and on-site or virtual field trips in the environment of MICE management. Moreover, more teaching resources, i.e., massive open online courses (MOOCs), including “Khan Academy” and “Coursea”, are recommended to be introduced into the class as a means of the integration of online and offline teaching.

Fourthly, the industry must take a proactive approach to help drive the reform in MICE education by taking the lead in the collaboration between industry and academia. The function of MICE education is to help academia focus on the employability skill sets that makes the graduates more capable and competitive in the employment market. The industry could first provide current undergraduate students with more entry-level and hands-on experience in the field of ICTs products and services. After that, a comprehensive collaboration plan should be established by all parties (i.e., the MICE companies, the colleges or universities, the students and other participants). In this plan, students are provided with more field-trip experience for ICTs-related facilities and resources in order to align curriculum system with MICE industry demands. As industry practitioners and experts provide timely feedback for practical teaching enhancement [37], internship, externship and other cooperative activities for ICTs skills are more likely to be implemented effectively, especially in cross-disciplines (MICE, information systems, and management) to accelerate career exploration for undergraduates [53]. Classroom visits by industry ICTs professionals and mentorships are vital opportunities of the industry-led development for MICE academic curricula and programs [38]. Comprehensive certifications (e.g., Certified Meetings Professional, CMP; Certified Special Events Professional, CSEP; Certified Government Meeting Professional, CGMP; Certification in Meeting Management, CMM; Certified in Exhibition Management, CEM) in MICE industry can lead to more continuing job opportunities, so certification programs are recommended for graduates to become MICE experts with a collection of knowledge, skills and capabilities. Professional certifications in connection with ICTs, such as Digital Event Strategist (DES) and Virtual Event & Meeting Management Certificate (VEMM), are also considered recommendable to enhance students’ practical skills for their future career. From the above, the creation of partnerships between academies and companies might be the fundamental to expand the knowledge of industry trends and promote the development of employability skills in MICE sector.

### Limitations and future research

This research, like other survey-based research, has limitations. Data in this research were self-reported by respondents, which may cause social desirability. Another limitation of this study is sample size. The collection of sample data is influenced and limited by COVID-19. The sample size is relatively small in the rigid sense, but still meets the requirement of minimum sample size. Most participants are undergraduate students, with less than one-year working experience, which might affect the representative of data sample. The specific context of Chinese MICE education in this study may also lead to limited generalization into other sectors and countries. However, the research itself might serve as a model for future employability skills of student-centered projects in MICE industry. It is meaningful and helpful to the industry and community, the university and college, and the students with a win-win-win situation.

To overcome the limitations, future research should be conducted on the perceptions regarding undergraduate- and graduate-level students, graduates who have been working in the field and MICE professionals to enlarge the sample size. Researchers may use multiple methods, such as in-depth qualitative interviews, in order to obtain a better understanding of employability skills in the viewpoints of MICE professionals. The exploratory nature of this study did not model the relationship among diverse partners. Hence, the existing data and conclusions could be explored and expanded with more participative interviews. A performance analysis with longitudinal research among stakeholders in MICE industry could be conducted to provide useful insights in regards to employability skills. Future studies may focus on the comparison among China and other countries, such as US and UK, to examine cross-cultural adaptation of the employability skills.

## Supporting information

S1 Data (XLS)
